# Hydatidiform Mole in a Patient With a Concern for Neoplasia: A Case Report

**DOI:** 10.7759/cureus.10319

**Published:** 2020-09-08

**Authors:** Indraneel K Prabhu, Amy Rosenbaum

**Affiliations:** 1 Obstetrics and Gynecology, Touro University Nevada College of Medicine, Las Vegas, USA; 2 Obstetrics and Gynecology, Women’s Health Associates of Southern Nevada, Las Vegas, USA

**Keywords:** hydatidiform, mole, gestational trophoblastic disease, choriocarcinoma, molar pregnancy

## Abstract

Molar pregnancy or a hydatidiform mole, also referred to as gestational trophoblastic disease, is an abnormal type of pregnancy, in which a potentially anomalous egg is abnormally fertilized resulting in a subsequently non-viable conceptus becoming an enlarged growth in the uterus with dangerous complications. These moles can occur as either complete or partial moles, each with its own unique features. In this article, we report a case of a pregnant woman who presented to her primary care doctor with the chief complaint of shortness of breath. Upon further questioning, she was found to have slight vaginal bleeding. Further workup of the unexplained bleeding revealed a small, yet concerning molar pregnancy, and led to our encounter with the patient on her operating day for a dilation and curettage. As these moles are somewhat rare occurrences, this case report aims to describe the condition, with a focus on management and outcomes.

## Introduction

Gestational trophoblastic disease (GTD) is defined as a nonviable conceptus causing a progressively enlarging uterine mass. It can initially present similar to a normal pregnancy: positive pregnancy test, physiologic changes of pregnancy, morning sickness, etc., especially when it is still small, making it easily missed. This is compounded by factors such as limited access to proper obstetric care, inconsistent follow-up, and presence of comorbidities that may confound the symptoms of molar pregnancy (as is the case with this patient). One feared complication of a molar pregnancy is progression to choriocarcinoma, a cancer that commonly metastasizes to the lungs, with some characteristic features detailed later. Although this cancer is quite chemosensitive and has a good prognosis, it is worth discussing molar pregnancy in further detail, so that signs may be recognized early, and an effective diagnostic and a timely treatment plan can be established to optimize outcomes, both mentally and medically, for the patient; this report aims to highlight these details while identifying obstacles to a proper diagnosis.

## Case presentation

This is a case of 37-year-old G0P0 who presented to a primary care doctor to establish care and for a routine well-visit, but did complain of worsening shortness of breath. She stated that she had had asthma ever since she was a child, well-controlled with an albuterol inhaler, but that for the past few months, her shortness of breath had been getting progressively worse, with only minimal relief with the inhaler. Outdoor exercise seemed to exacerbate her symptoms, and she generally felt “normal” while sitting. Although the symptoms were worse than her usual baseline, the patient described the “discomfort” severity as ~4/10. She did not have any associated chest pain, and the review of systems was negative for fever, chills, diarrhea, musculoskeletal pain, and dysuria but was positive for headache, mild nausea and vomiting, and slight urinary incontinence and frequency. Her family history was noncontributory. Her surgical history is significant for tonsillectomy some time in her early childhood, as well as an elective C-section from a personal request. The patient's social history was noncontributory. She was currently sexually active with her husband, and they did not use any barrier contraceptive method. The patient had no known drug allergies, and she used to take ibuprofen for painful menstrual cycle each month, but told her primary that she had stopped three months prior, as her periods had stopped completely. Further questioning revealed that the patient was pregnant, per a home pregnancy test, and it came to light that she had not had any prenatal or antenatal care. At this point, the patient also mentioned that she had bouts of irregular bloody-brown vaginal discharge that occurred in unpredictable patterns for the past three months, previously occurring regularly in 24-day cycles. This prompted a focused obstetric and gynecologic history and physical examination, with a prompt referral to an obstetrician/gynecologist.

At her first visit, the routine tests were run, and in addition, a speculum exam was done, along with a pap smear, to fully establish care. Due to her complaint of the bloody-brown discharge, a transvaginal ultrasound was also performed to assess fetal well-being, and was further supplemented with a serum pregnancy test to confirm the initial positive result from the home pregnancy test. On physical exam, the patient had stable vitals. Lungs were mostly clear to auscultation bilaterally, with some wheezing noted on both inspiration and expiration, without rales or rhonchi. An abdominal exam showed mild suprapubic tenderness with distention. The uterus was palpable and was smooth without irregularities, and was consistent with a 20-week gestation (palpable at the umbilicus), though her last menstrual period was 16 weeks prior. The speculum exam showed a closed nulliparous cervix with no discharge or bleeding present in the vaginal vault. The rest of the exam was unremarkable.

Laboratory results (initial antenatal) are presented in Table 1.

**Table 1:** Initial antenatal laboratory results RBC: red blood cell; WBC: white blood cell; Hgb: hemoglobin; hCG: human chorionic gonadotropin; VDRL: venereal disease research laboratory test; CBC: complete blood count; PCR: polymerase chain reaction; Hbs: hepatitis B surface antibody; Hbc: hepatitis B core antibody; HbsAg: hepatitis B surface antigen; hpf: high power field

Test	Value
Urinalysis + dipstick	Creatinine 0.9 mg/dL, urea nitrogen 19 mg/dL, negative nitrites, negative leukocyte esterase, specific gravity 1.011, no casts, <3 RBC per hpf, <3 WBC per hpf, negative for protein, mild glucosuria
Urine culture	Negative for bacteria
Rh(D) antibody screen	(-) for antibodies
Hepatitis B	(+) anti-Hbs antibody, (-) HbsAg, (-) anti-Hbc antibody
Rubella	Immune
Varicella	Immune
HIV	Negative
Syphillis	(-) VDRL
Chlamydia	(-) PCR
Serum beta-hCG	192,000 mIU/mL
CBC	RBC count: 4.0 million cells/mcL, Hgb: 11.5 g/dL, Hct: 43.5%, WBC count: 9500 cells/mcL, platelet count: 300,000/mcL

The transvaginal ultrasound report described a “mass within the uterus with a snowstorm appearance” and noted that “no viable intrauterine pregnancy was seen, with no sign of fetal heart tones that one would expect given the gestational age and serum beta-human chorionic gonadotropin (HCG) levels”. The report also noted “bilateral cystic masses seen in both adnexa, without necrosis of ovaries”. The masses were hypoechoic, fluid filled, and were without septations or calcifications, and measured approximately 3.5 cm in diameter on each side. Due to suspicion for molar pregnancy, a histological sample was obtained from the mass to confirm the diagnosis. Histopathology showed cells with marked cytological atypia, diffuse hydropic villi enlargement, with hyperplasia of the trophoblastic cells within the sample. In addition, many of the hydropic villi exhibited a sort of fibrillary separation within the core, leading to the appearance of being widely separated from one another, described as having a “cistern formation”. Her bilateral adnexal cystic masses were very likely benign theca lutein cysts that resulted from the markedly high levels of beta-HCG within the bloodstream, causing hyperplasia of the theca interna cells within the ovary, and the patient was advised that these would likely resolve with treatment of the molar pregnancy itself. Given the risk for choriocarcinoma, which most commonly metastasizes to lung, and her worsening shortness of breath, a chest X-ray was obtained. The results were unremarkable and thus no further imaging was pursued.

With the diagnosis of molar pregnancy confirmed by histology, the first priority was to evacuate the mole via dilation and curettage (D&C). An incomplete evacuation could lead to persistent GTD from retained molar tissue. The next step was to monitor that all of the trophoblastic tissue that had proliferated was truly eradicated; beta-HCG monitoring was started, comparing each obtained value to the baseline pre-treatment value of 192,000 mIU/mL. Oral contraceptives were initiated to ensure that any beta-HCG being monitored would be from remnant mole alone, and not a new intrauterine or ectopic pregnancy. The patient was advised on this course of action, and recommended to follow up every week, and she agreed to this plan.

## Discussion

A molar pregnancy, also known as hydatidiform mole or GTD, is an abnormal, nonviable pregnancy that results from the anomalous fertilization of a potentially empty ovum by one or more sperm. The resulting mole is a benign but rapidly growing mass that is cystic, and can mimic the signs and symptoms of normal pregnancy quite early on, and can therefore go undetected. Once diagnosed, the patient must be treated quickly, as the complications associated with this condition can be life threatening, and there is a risk of malignant transformation to choriocarcinoma.

Partial mole

Normal fertilization is the process by which one single sperm fuses with one single egg, or oocyte, leading to a zygote. This process occurs in the ampulla of the Fallopian tube, and over time, the zygote travels down the Fallopian tube to reach the uterus, where it will implant, leading to a normal pregnancy. Normally, the sperm enters the oocyte through an acrosomal reaction to penetrate the zona pellucida (ZP) and implants. At this point, an important step known as the cortical reaction takes place, with cortical granules in the secondary oocyte releasing into the ZP, causing glycoprotein ZP2 to undergo hydrolysis. The extracellular matrix within the ZP is now known as ZP2f [1], and is tightly cross-linked and impermeable to entry by any new sperm - an important mechanism to prevent polyspermy. Failure of this process can lead to more than one sperm fertilizing the egg and leading to subsequent secondary meiosis with an eventual partial mole. Because partial moles are resultant from (usually) two sperm fertilizing one complete egg (incidence ~3 per 1000 pregnancies) [2], the karyotype generally seen is 69XXX or 69XXY. Since the egg did contain its normal genetic material (compare to section "Complete mole"), there will be some fetal parts evident, but not enough to see a living conceptus or any viable organism. More often than not, an amniotic sac will also form, leading to a potential confounder when trying to diagnose this condition on ultrasound, depending on the user’s skill. With the presence of this somewhat normal tissue, the amount of villous edema that is seen is focal in nature, and less widespread than that of a complete mole. The amount of trophoblastic tissue available to proliferate is less so than that of a complete mole, and as such, the uterus does not rapidly enlarge, and without a completely viable fetus, is much smaller than the gestational age would suggest at a given point in time. The beta-HCG levels in a partial mole are not as markedly high as in a complete mole, and as such any medical sequelae resulting from elevated beta-HCG are generally not seen. These include hyperemesis gravidarum (HG), hyperthyroidism, theca lutein cysts, and/or preeclampsia with severe features. Though present, the theoretical risk of progression of a partial mole to full-on choriocarcinoma is less than 2%-3% [2]. There is, however, a 5% risk of persistent GTD, where some of the mole is retained within the uterus even after adequate treatment. Partial moles commonly result in uterine sizes much smaller than what is expected given a particular gestational age, as there is a lack of proliferation of normal fetal tissue.

Complete mole

There are two main mechanisms by which a complete mole (~1-2 per 1000 pregnancies) can arise [2]. About 85%-90% of the time, normal fertilization occurs, but soon after, the maternal genetic material is destroyed and expelled. The single sperm then duplicates itself, giving rise to the majority monospermic, androgenetic 46XY genotype of the complete mole. The other 5%-10% of the time, two sperm are able to fertilize a normal oocyte, with subsequent destruction/expulsion of the maternal genetic material, leading to the minority dispermic androgenetic 46XY genotype of the complete mole [3]. The overexpression of paternal genes through these mechanisms causes the proliferation of synctiotrophoblasts, and it has been hypothesized that sperm have the genetic code allowing them to proliferate as much as possible to have the highest chance of producing a successful fertilization [4]. The oocyte genes code to maximize the chance of having the successful development of a normal conceptus once fertilization has occurred. There have also been theories supporting the loss of maternal genetic material within an oocyte prior to penetration by any number of sperm, but no data has been found to support these claims. Although triploid karyotypes are in the realm of a genetic possibility for a complete mole, by far the vast majority are diploid with the 46XY genotype. With the maternal chromosomal material being lost in the “fertilization” process, there will not be any fetal parts present, without the normal zygotic tissue, and thus, no amniotic sac will be evident either. The lack of any normal fetal tissue allows for more space for the trophoblastic proliferation of tissue, as evidenced by diffuse proliferation of this tissue, leading to the classic “cistern formation” with widespread hydropic villi throughout the resultant uterine mass. The villi within a complete mole, as seen histologically, do not have a clustered appearance, as the foci of villi in a partial mole would suggest, but rather have a swollen sort of appearance due to the widespread edema within the mass itself.

Complete moles secrete high levels of beta-HCG, much higher than what would be expected of a normal pregnancy, at a given point in time. This proves to have an important role as a tool when trying to diagnose a complete mole. The rapid rise in the beta-HCG level can differentiate this from a normal pregnancy, and is responsible for the potential complications such as hyperthyroid features, HG, theca lutein cysts, and/or preeclampsia with or without severe features. There is also a 6%-32% chance of progression to malignant choriocarcinoma, and about a 12%-15% chance of progression to persistent GTD [5]. These all depend on many patient factors, but must be carefully monitored, as they can all prove to be damaging, or even fatal. Commonly, a complete mole results in a uterus that is much larger in size than what would be expected given a certain gestational age, and must always be included in the differential diagnosis when this is found. There are a few mechanisms that have been proposed as to the development of the proliferative and rather invasive nature of complete moles, namely, placental regulatory factors. Uteroplacental regulators have been studied as a means of communicating between maternal cells and trophoblastic cells in the developing placenta and fetus to maintain normal pregnancy and development. During the first trimester, a relatively hypoxic environment promotes the development of the trophoblast while avoiding the toxicity of oxygen radicals through the reduction of oxidative stress [4]; this allows for the proliferation to not go unchecked and prevents extravillous tissue from becoming invasive. More specifically, the lack of oxygen during the first trimester leads to the upregulation of hypoxia-inducible factor 1-alpha, which in turn activates transforming growth factor (TGF)-beta. TGF-beta, along with another molecule called decorin, is postulated to be responsible for negatively regulating the proliferation and migration of trophoblastic tissue [4]. The rapidly growing tissue within a complete mole potentially has resistance to this negative downregulation of growth by TGF-beta, and is one growingly supported hypothesis for how GTD develops [5]. After around nine weeks of gestation, the partial pressure of oxygen (pO_2_) of the feto-maternal environment increases to allow for proper spiral artery development and proper perfusion of the placenta, an expected phenomenon [5]. More research in this regard is needed before any cause-and-effect relationships can be definitively drawn.

Risk factors

Although the causes of molar pregnancy and hydatidiform moles have not been clearly defined, there are several risk factors that have been proposed and studied. One of the most important factors is having a past history of a complete mole. With a positive history, the risk of developing a complete mole in a subsequent pregnancy is 1%-1.5%, a seemingly small, yet significant number when one considers the implications [6]. This association with a previous occurrence of molar pregnancy has not been linked to partial moles, and the reason behind this has not been well-established. Advanced maternal age is another significant risk factor, with women over the age of 35 being at a slightly elevated risk, and those over the age of 45 having over 2.5 times the risk of that in a 35-year-old pregnant woman [6]. Interestingly, however, the same risk is not seen for mothers at the opposite extreme (young age), for reasons not yet established in the literature. A 2018 study analyzing these risk factors found them to be statistically significant, with P = 0.001, for advanced maternal age, and P = 0.05, for a previous mole [6]. In addition, a molar pregnancy towards the end of the first trimester was a strongly associated risk factor, with P = 0.04. These studies also showed that oral contraceptive use did not reduce or increase the risk of mole formation upon cessation of the contraceptive.

An interesting link was also tied to the mother’s blood group. A study in 1985 examined the incidence of having both partial and complete moles in mothers with varying blood types. It was found that having a Type A blood group conferred a relative risk (RR) = 1.4, and having Type AB conferred an RR = 2.310 [7]. After stratification for potential confounding by paternal blood typing, both A and AB blood types still showed a statistically significant increased risk for having a molar pregnancy. Interestingly, Type O and Type B blood groupings did not have this same risk increase for molar pregnancy. Researchers have purported potential differences in immunity factors circulating in the bloodstream of mothers with these different blood groups, but a definitive cause has not yet been established. A study conducted in 1987 also found that living in North America seemed to be a protective factor against the development of a mole, be it partial or complete [1]. Living in Europe conferred a slightly higher risk, but was not statistically significant; however, living in Asia had an RR of 1.9 (P < 0.05) for mole development [1]. One theory suggested was the lack of access to adequate healthcare in many of the countries that were studied. However, the same study posited that it was not a lack of medical resources, but rather a deficiency in vitamin A and carotene in the world outside of North America that caused these findings. Though vitamin A in excess can be quite teratogenic, women who consumed a diet with carotene levels slightly higher than the daily recommended value (DRV) had an RR of 0.6 (P < 0.02), for molar pregnancy [1], showing a statistically significant protective benefit. The reverse also proved to be true: a deficiency of carotene showed a statistically significantly elevated risk of GTD.

A few other less studied risk factors have also been put forth by researchers, and much less data on these claims exists, but for completeness will be briefly discussed here. A 2017 study sought to analyze paternal factors potentially increasing the risk of a molar pregnancy [8]. Of all the factors studied, the only statistically significant finding was the father having a job that required physical labor involving dust and soil, with an odds ratio (OR) of 18.2 (P < 0.001). The researchers involved noted in their discussion that further studies would need to examine why this relationship existed, as the number of confounding variables was indeterminate at the time of study.

Diagnosis

As in the case of most clinical cases, a thorough history and physical exam should be considered the utmost gold standard when diagnosing and managing a patient. This became even more evident in the case of this patient, where the chief complaint led to a nearly nonexistent index of suspicion for a molar pregnancy. The patient volunteered information about her pregnancy status as well as bloody vaginal discharge only after being prompted through a thorough interview, and led to the subsequent workup. This workup was extensive, as the patient had never established prenatal or antenatal care, and bleeding from the vagina was concerning for threatened or missed abortion. As the speculum exam was normal in this patient, the next step, in addition to tests listed in Table 1, was a transvaginal ultrasound and a serum beta-HCG level, used to properly confirm and date the pregnancy. Given the patient’s account of her last menstrual period, the finding of a markedly elevated beta-HCG level, and uterine size significantly larger than expected given her gestational age, a list of differentials began to form, which included partial and complete hydatidiform moles. The transvaginal ultrasound, which uses a transducer, or “wand” to send sound waves throughout the pelvis, shows a characteristic “bunch of grapes” appearance or “snowstorm” pattern, nearly pathognomonic for GTD.

A definitive answer is via biopsy and histological examination, and together with the clinical snapshot of the patient makes for the diagnosis. Histology can not only confirm the diagnosis of a mole, but can also quickly differentiate between a partial and a complete one. A partial mole is generally small, and contains less than 250-300 cc of trophoblastic tissue. As the maternal DNA is still present, there is a somewhat normal amount of fetal tissue present, with an associated placenta, and can be seen under a microscopic examination. With the presence of fetal parts, there is less space for trophoblastic tissue to unnecessarily proliferate, and this is seen in the form of foci of edematous villi that stay contained without becoming widespread. There is little to no cytological atypia within a partial mole. Complete moles, however, have no normal tissue and no fetal parts, giving a large space (approximately 500-600 cc of tissue) to proliferate; this is evidenced by the widespread, diffuse enlargement of hydropic villi and edema. The edema pushes aside many cellular structures and contributes to the classic “central cistern” formation. Complete moles have a much higher risk of malignant transformation, owing much of this risk to the marked cytological atypia seen under a microscope.

Complications

Most, if not all, of the complications from GTD arise due to the markedly elevated beta-HCG levels. At physiologically appropriate levels, beta-HCG maintains pregnancy by upkeep of the corpus luteum, allowing for progesterone secretion until around week 10, when the placenta can make its own. It is also responsible for stimulating Leydig cells to produce testosterone in the male fetus, steroidogenesis in the placenta and adrenal glands, and the maternal thyroid to continue normal functioning during pregnancy. When these levels stay persistently elevated and unchecked, problems begin to arise.

Theca Lutein Cysts

Theca interna cells of the ovary are normally responsible for secreting androgens and estrogens via pregnenolone intermediates, and are responsive to beta-HCG during pregnancy. When beta-HCG levels are persistently and markedly elevated, these cells continue to respond and undergo hyperplasia from the sustained stimulus. This rapid enlargement is most commonly bilateral and creates adnexal masses known as theca lutein cysts. These are mostly benign, usually found incidentally on transvaginal or abdominal ultrasound when examining the ovaries, and are simple, fluid-filled cysts. While most are asymptomatic, patients can complain of feelings of adnexal fullness or peritoneal irritation if the cysts rupture. Due to the mass effect of cysts greater than 3 cm, there is also the risk of ovarian torsion, which would be a medical emergency, but is rare with these cysts in particular, as they rupture before this can happen. It has been documented that women who smoke are at a higher risk of having larger theca lutein cysts as well [9]. Treatment is generally supportive as the cysts resolve once the levels of beta-HCG come back to baseline, which is usually after the pregnancy ends.

Hyperthyroidism

Beta-HCG normally stimulates the maternal thyroid to meet the increased metabolic demand of having a growing fetus, but lies on a fine balance. Too much beta-HCG can cause hyperthyroidism, ranging from mild symptoms to overt thyrotoxicosis. The basis for this is the structural homology between these two molecules. Although their alpha subunits differ, the beta subunits of both HCG and thyroid stimulating hormone (TSH) share 85% sequence identity, which explains why beta-HCG has such a high, let alone any, thyrotropic activity. HCG’s beta subunit in addition contains a 31 amino acid extension on the carboxy terminal that has been theorized to attenuate how much thyrotropic activity the molecule actually has [9]. A study has shown that a recombinant version of HCG that lacks this amino acid sequence at the carboxy terminal has nearly the same activity level at the TSH receptor as TSH itself [10]. Normally in pregnancy, increased estrogen levels lead to increased thyroid binding globulin (TBG) production, and thus, the “total” thyroid hormone levels are increased, as the thyroid gland pushes more hormone out as more is bound to TBG. However, the measurement of “free” T3 and T4 will still be normal, and no symptoms of hyperthyroidism are seen. The symptoms that should prompt further investigation include sweating, heat intolerance, diarrhea, tremor, anxiety, palpitations, weight loss (especially in pregnancy, where the norm would be weight gain), etc. With the most common cause of this being Grave’s disease, a workup for TSH receptor stimulating antibodies should be undertaken, prior to suspecting trophoblastic tumors. Thyroid storm should be suspected with hyperthermia, new onset atrial fibrillation, confusion, coma, or severe restlessness, and should be managed with supportive measures and medications immediately. As with theca lutein cysts, the risk of hyperthyroidism and its sequelae is effectively eliminated once the beta-HCG stimulus resolves with the evacuation of the tumor.

Hyperemesis Gravidarum

Morning sickness, or a mild amount of nausea/vomiting in pregnancy, is considered a normal effect of beta-HCG, but can pose dire risks for the mother when this becomes persistent. HG is characterized by extreme and persistent nausea and vomiting that can lead to dehydration, weight loss, and life-threatening electrolyte imbalances, and is considered a medical emergency, with an incidence of about 1.5%-3% worldwide [11]. This condition typically occurs before the 20th week of pregnancy, since the first trimester is when beta-HCG levels are the highest. Risks include a prior history of HG, family history of HG, twin gestations (higher HCG levels than a single pregnancy), and hydatidiform moles. Although generally a diagnosis of exclusion, loss of 5% of pre-pregnancy body weight combined with ketosis from dehydration and inability to consume calories precludes further testing and warrants hospitalization to stabilize the patient. Laboratory values generally show metabolic alkalosis resulting from stomach acid loss through continuous vomiting, and vitamin deficiencies become common with prolonged vomiting. Thus, management involves supportive measures with IV fluid administering to maintain hydration status and blood pressure stability, repletion of vitamins such as thiamine and pyridoxine to prevent Wernicke’s encephalopathy or peripheral neuropathy, and anti-emetics combined with small-portioned dry foods to attenuate emesis. Should the above management not prove sufficient, complications of severe HG can be seen in the form of anemia, hyponatremia and its sequelae, Mallory-Weiss tears from continuous vomiting, hypoglycemia, and malnutrition. These can pose risks to the fetus as well, in the event of a normal pregnancy, in the form of slow growth and failure to thrive. In our patient’s case, she had mild nausea and vomiting, and none of the other signs of HG, so further workup in this regard was unnecessary.

Preeclampsia

Preexisting hypertension can be exacerbated by pregnancy, and can lead to growth restriction for the fetus, along with a host of maternal complications. When a pregnant mother has no preexisting hypertension, but develops it after 20 weeks of gestation, this is known as gestational hypertension. If the mother is also found to have new onset proteinuria with signs of end organ damage, this now becomes categorized as preeclampsia. One mechanism by which preeclampsia occurs is through placental hypo-perfusion leading to a hypoxic state, causing constriction of spiral arteries. Although there are many ways this can occur, the invasion and overgrowth of trophoblastic tissue can compress spiral arteries recreating this same hypoxic environment, thus either directly causing, or exacerbating existing preeclampsia [11]. Risks for developing preeclampsia include prior history of preeclampsia, preexisting hypertension, gestational hypertension, kidney disease, tobacco use, advanced maternal age, obesity, multiple gestations, and diabetes mellitus. The treatment for preeclampsia without severe features is the management of blood pressure and delivery at term (37 weeks). Severe features of preeclampsia include systolic blood pressure greater than 160 mm Hg or diastolic greater than 110 mm Hg, thrombocytopenia, elevated liver enzymes with associated right upper quadrant pain, new-onset pulmonary edema, elevated creatinine, visual changes, or cerebral changes/deficits. One feared outcome is progression to full-blown eclampsia, which involves life-threatening seizures, so recognition and management of this condition is vital to positive maternal and fetal outcomes. The treatment for preeclampsia with severe features is strict management of blood pressure with plans for delivery at 34 weeks. Most, if not all, cases resolve with delivery. There has been evidence to show that giving low-dose aspirin starting at 12 to 16 weeks of gestation in high-risk patients can improve risks of developing preeclampsia [12]. In the case of our patient, she was normotensive and did not show any signs of end organ damage or proteinuria, so close monitoring was sufficient in this regard.

Choriocarcinoma

Malignant transformation of a complete mole can occur in 6%-32% of cases [4]. Half the occurrences of choriocarcinoma arise from a preexisting complete hydatidiform mole; spontaneous abortions make up 20% of the remaining cases and ectopic pregnancies account for about 2.5%-3% of cases. Normal pregnancies account for approximately 25%-30% of cases as well [4]. Therefore, a major risk factor is trophoblastic disease and, if recognized early, can be crucial to positive outcomes for the mother. Stage I disease is limited to the uterus, Stage II is spread to the cervix and vagina, Stage III is single-organ metastasis (most commonly lungs), and Stage IV is multi-organ metastasis (usually liver and brain). As this tumor is the result of chromosomal material of paternal origin, like most male-specific tumors, it is quite chemosensitive. While D&C can prove effective for moles, the presence of malignant tissue calls for a more thorough eradicative approach. For low-risk moles, generally Stage I, methotrexate has proven adequate. By inhibiting dihydrofolate reductase, methotrexate is able to inhibit DNA synthesis, and rapidly dividing cancer cells are unable to effectively do so any longer. A popular chemotherapeutic regimen for more advanced disease is the etoposide, methotrexate, actinomycin, cyclophosphamide, and vincristine (EMACO) combination regimen. A 1991 study aimed to demonstrate the efficacy of this regimen, and found that when compared to those undergoing a different regimen for trophoblastic disease, the EMACO regimen yielded an 82% remission rate, following the cohort to demonstrate an 85% survival rate [13]. For those acquiring drug resistance, it was shown that the addition of cisplatin could salvage 82% of the remaining subjects without surgery, whereas salvage surgery alone could save 87%, but came with its own risks. The toxicity and drug interactions of these chemotherapy agents are quite well known and predictable, and the benefits of using them to treat advanced choriocarcinoma almost always outweigh the risks of doing so. Our patient did not show signs of malignant transformation, and therefore did not require more advanced treatment. When there is a high index of suspicion for malignant transformation, it must be considered metastatic until proven otherwise. Choriocarcinoma has a tendency to spread to the lungs most commonly, and less so to the brain. In the case of our patient, though she had asthma to possibly explain her shortness of breath, a spread to the lungs of potential choriocarcinoma had to be ruled out, with the initial step being a chest X-ray. If there were truly metastases causing her dyspnea, they would be evident as classic “cannonball metastases”, which was not the case for our patient. The presence of findings on X-ray would then prompt an immediate high-resolution CT scan of the chest to localize the lesions and set up a treatment plan, and serves as a baseline to track therapeutic progress. CT scans are also important when staging the spread to less common regions of the body. Brain metastasis has been well-documented for this disease, and generally presents with new-onset headaches, visual changes, neurologic deficits, and seizures that prompt an imaging workup of the area. CT and MRI can be and have been used, depending on availability and stability of the patient. It is important to note that these imaging studies should be used as a pre-treatment baseline, with close follow-up and re-imaging to ensure eradication of malignant cells.

Treatment

The centerpiece of treatment of molar pregnancy is removing the mole from the body in its entirety. The two methods utilized are uterine evacuation in the form of a D&C, or a complete hysterectomy, each with its pros and cons. A D&C is preferred in younger women who prefer to maintain their fertility, and is recommended for women not of advanced age, and who have partial moles. When the risk of progression to gestational trophoblastic neoplasia is increased, as in the case of age greater than 40 or in cases of a complete mole, then a hysterectomy is the preferred choice [14]. Although hysterectomy nearly eliminates the risk of local neoplasia, some women already have occult metastasis that goes undetected, and must be considered if symptoms arise after the uterus has been removed. This is supported by a study that looked at malignant transformation of moles and it was found that in complete moles removed by a D&C, there was a 55% recurrence rate due to retained tissue, with a 0% recurrence rate in those patients who underwent total abdominal hysterectomy [15]. After our conversation with our patient, a D&C was decided on, as the patient still wanted to preserve her fertility for some years, and did not want to undertake the risks of having an invasive surgery if she could avoid it. Medical management alone should not be used, as a study showed that nearly 26.2% of cases managed solely with medication required a surgical consult within the next five years, compared to only 3.8% of initial surgically managed patients [15].

Prior to surgery, patients with various complications resulting from the molar pregnancy should have them effectively controlled. For instance, overt hyperthyroidism should prompt administration of a beta blocker such as propranolol to prevent surgery-induced thyroid storm. Preeclampsia with or without severe features should have tightly controlled blood pressure throughout the surgery, and magnesium sulfate should always be on hand in the case of eclampsia-related seizures. In the case of a partial mole, traces of fetal blood may be present within the normal portion of the placenta, and this could result in feto-maternal blood mixing; the clinician should take care to administer anti-D immunoglobulin should the mother be RhD negative (seen in this patient with a negative initial antibody screen) to prevent sensitization and alloimmunization in future pregnancies. With a D&C, disruption of the mole can cause quickly progressive hemorrhage, and this can be more so than that of a normal pregnancy. For this reason, oxytocin is given soon after the anesthesiologist sedates the patient, to constrict the spiral arteries and prevent bleeding; blood should be typed and cross-matched, ready to transfuse the patient if needed. This can be augmented with uterine massage transabdominally during the evacuation process. After the D&C is complete, a final check of the uterine cavity should be undertaken to ensure a complete removal of trophoblastic tissue, and the removed mass should be sent to pathology for examination. In the case of uncontrollable hemorrhage, one can consider immediately moving to hysterectomy, or less commonly, embolization of the uterine arteries. Hysterectomy is generally done laparoscopically to improve healing time, but runs the risk of injuring the ureters, as they are in close proximity to the surgeon’s workspace. With a transabdominal approach, symptomatic theca lutein cysts that can commonly be present can be drained or removed to prevent advancement to a torsion.

Follow-up of the patient postoperatively to monitor beta-HCG levels is a crucial part of appropriately managing the patient. The beta-HCG level should return to baseline, while on contraception, over a period of some months, had the operation been successful at evacuating all traces of the trophoblastic tissue. The beta-HCG levels should be monitored weekly until the levels are undetectable or reach an acceptable plateau, with protocol described as the following: undetectable HCG levels for three consecutive weeks. If either of these criteria are met, then continued monitoring monthly is done for three months, and then no further if levels stay low; for a partial mole, monthly monitoring is done for one month and then done no further. For our particular patient, her prior medical history included migraines and a contraindication to estrogen-containing contraceptives. Intrauterine devices are relatively contraindicated, as they can worsen bleeding and place the patient at risk of uterine perforation, should the mole have invaded the uterine wall. As such, the patient was counseled on starting a progestin-only mini pill, supplemented by condom use, and followed the described protocol.

Outcomes

GTD with mole formation is a relatively rare occurrence, and has complications that warrant concern and close monitoring, but generally has a good prognosis with early intervention. When our patient was given her diagnosis of a complete hydatidiform mole, her biggest concern was preservation of her fertility, evidenced by her aversion to hysterectomy, and risk for other gestational complications in the future. A review between 1965 and 2013 by the New England Trophoblastic Disease Center [16], which followed 667 pregnancies in women who had been treated for molar pregnancy, found statistically significant outcomes listed in Table 2.

**Table 2:** Outcomes following a molar pregnancy Data is taken from [16].

Significant outcomes	
Live term births	66.9%
Spontaneous abortions	18.3%
Premature preterm deliveries	6.6%
Other abortion unspecified	4.2%
Stillbirth	1.5%
Repeat molar pregnancy	1.5%
Ectopic pregnancy	1.0%

For our particular patient, other than her complete mole, her only true risk factor for any sort of gestational complications was her advanced maternal age, and as it stood, she had a very good chance of having a normal pregnancy with proper prenatal and antenatal care. Watchful management of the other listed complications was appropriate. A thorough staging workup for choriocarcinoma would not be required for most people, but extra precautions were taken with this patient who presented with shortness of breath, later found to be simple asthma exacerbation from the cold and dry winter air in the desert environment. Adding inhaled corticosteroids to her short-acting beta agonist inhaler helped curb these extra symptoms, which was a reassuring sign on her path to recovery.

The progression to choriocarcinoma from a complete mole lies between 6% and 32%, a wide and disconcerting range for those with GTD. However, for those diagnosed with malignant transformation, the prognosis still remains very good. The use of chemotherapy does not pose any increased risk of fetal malformation from baseline. For Stage I choriocarcinoma, single-agent chemotherapy cured 83% of patients in an analysis between 1985 and 2013 [12], with the other 17% also achieving complete remission with additional chemotherapy or surgery. In Stage II through IV choriocarcinoma, all patients were cured, but required surgery and additional chemotherapy from the base management algorithm. Recurrence rates of neoplastic disease are listed at 5%-10% of cases, with those at the higher end of the spectrum being due to large initial tumor burden, delayed care, and patients not adherent to therapy [17]. It is important to note that a PET scan should be considered in recurrent cases, to differentiate between new active tumors and the fibrotic plaques resulting from past tumors and chemotherapy induced fibrosis.

Our patient showed strict compliance with treatment guidelines, and attended all appointments scheduled for her. She reported taking her progestin-only mini pill every day at the same time upon waking up, and continued to be watchful for any relapse of symptoms or new symptoms that arose. At her two-week follow-up, beta-HCG levels had reduced drastically, and showed a promising downward trend, reducing the likelihood of there being any remnant tissue or malignant tissue, though that risk was not zero. The patient was advised that after her beta-HCG levels stabilize, it was in her best interest to not try to become pregnant for one year, to allow the uterus and placenta to normalize, best increasing her chance for a normal pregnancy while reducing any of the negative outcomes listed in Table 2. It has also been recommended that a beta-HCG level should be checked six weeks postpartum in future pregnancies, for mothers who have had a history of GTD.

## Conclusions

Molar pregnancies are rare occurrences, but the index of suspicion should be high when a patient presents with unexplained vaginal bleeding after a positive serum pregnancy test, symptoms of hypothyroidism, hyperemesis gravidarum, dyspnea, or uterine size discrepancy given a particular gestational age. Suspected moles can be mostly ruled in with a transvaginal ultrasound; however, definitive diagnosis requires histopathological evaluation after an adequate biopsy. After diagnosis, surgical management is the cornerstone of treatment, either in the form of a D&C to evacuate the uterus of trophoblastic contents, or a hysterectomy. Fortunately, for our patient, the evacuation of the mole and subsequent beta-HCG testing while on contraception were without further complications.

## References

[1] Wilson JH, Hunt T (2002). Molecular Biology of the Cell: A Problems Approach. https://books.google.co.in/books/about/Molecular_Biology_of_the_Cell.html?id=BFv6QwAACAAJ&redir_esc=y.

[2] Cavaliere A, Ermito S, Dinatale A, Pedata R (2009). Management of molar pregnancy. J Prenat Med.

[3] Vassilakos P (2020). Pathology of molar pregnancy. In Reproductive Health.

[4] Soper JT, Mutch DG, Schink JC, for the American College of Obstetricians and Gynecologists (2004). Diagnosis and treatment of gestational trophoblastic disease: ACOG Practice Bulletin No. 53. Gynecol Oncol.

[5] Candelier JJ (2016). The hydatidiform mole. Cell Adh Migr.

[6] Mulisya O, Roberts DJ, Sengupta ES (2018). Prevalence and factors associated with hydatidiform mole among patients undergoing uterine evacuation at Mbarara Regional Referral Hospital. Obstet Gynecol Int.

[7] Parazzini F, La Vecchia C, Franceschi S, Pampallona S, Decarli A, Mangili G, Belloni C (1985). ABO blood-groups and the risk of gestational trophoblastic disease. Tumori.

[8] Shamshiri Milani H, Abdollahi M, Torbati S, Asbaghi T, Azargashb E (2017). Risk factors for hydatidiform mole: is husband’s job a major risk factor?. Asian Pac J Cancer Prev.

[9] Yoshimura M, Hershman JM (1995). Thyrotropic action of human chorionic gonadotropin. Thyroid.

[10] Marx H, Amin P, Lazarus JH (2008). Hyperthyroidism and pregnancy. BMJ.

[11] Jennings LM, Krywko D (2020). Hyperemesis Gravidarum. StatPearls [Internet]. https://www.ncbi.nlm.nih.gov/books/NBK532917/.

[12] Vitoratos N, Hassiakos D, Iavazzo C (2012). Molecular mechanisms for preeclampsia. J Pregnancy.

[13] Newlands ES, Bagshawe KD, Begent RH, Rustin GJ, Holden L (1991). Results with the EMA/CO (etoposide, methotrexate, actinomycin D, cyclophosphamide, vincristine) regimen in high risk gestational trophoblastic tumours, 1979 to 1989. Br J Obstet Gynaecol.

[14] Carey L, Nash BM, Wright DC (2015). Molecular genetic studies of complete hydatidiform moles. Transl Pediatr.

[15] Berkowitz RS, Cramer DW, Bernstein MR, Cassells S, Driscoll SG, Goldstein DP (1985). Risk factors for complete molar pregnancy from a case-control study. Am J Obstet Gynecol.

[16] Vargas R, Barroilhet LM, Esselen K, Diver E, Bernstein M, Goldstein DP, Berkowitz RS (2014). Subsequent pregnancy outcomes after complete and partial molar pregnancy, recurrent molar pregnancy, and gestational trophoblastic neoplasia: an update from the New England Trophoblastic Disease Center. J Reprod Med.

[17] Lurain JR, Elfstrand EP (1995). Single-agent methotrexate chemotherapy for the treatment of nonmetastatic gestational trophoblastic tumors. Am J Obstet Gynecol.

